# Is There a Place for Adjuvant Chemotherapy in the Treatment of Locally Advanced Cervical Cancer?

**DOI:** 10.3390/curroncol29080415

**Published:** 2022-07-23

**Authors:** Dora Čerina, Tihana Boraska Jelavić, Matea Buljubašić Franić, Krešimir Tomić, Žarko Bajić, Eduard Vrdoljak

**Affiliations:** 1 Clinical Hospital Center Split, School of Medicine, Department of Oncology, University of Split, 21 000 Split, Croatia; dora09cerina@gmail.com (D.Č.); mateabuljubasic@yahoo.com (M.B.F.); 2 Department of Health Studies, University of Split, 21 000 Split, Croatia; tihana_boraska@yahoo.com; 3 Department of Oncology, University Hospital Mostar, 88 000 Mostar, Bosnia and Herzegovina; drkresimirtomic@gmail.com; 4 Research Unit “Dr. Mirko Grmek”, University Psychiatric Hospital “Sveti Ivan”, 10 000 Zagreb, Croatia; zarko@biometrika.hr

**Keywords:** uterine cervical neoplasms, locally advanced cervical cancer, concurrent chemoradiation, adjuvant chemotherapy

## Abstract

Findings on the efficacy of adjuvant chemotherapy (ACT) of locally advanced cervical cancer (LACC) after the concurrent chemoradiation (CCRT) therapy were inconsistent, and the OUTBACK trial was expected to shed some light regarding the topic. Its results on ACT in LACC were negative, with the conclusion of not to use it. The objective of this review was to present the inconsistencies of previous studies, along with the OUTBACK trial in more detail, and to rethink whether its results provide an unambiguous and definite answer to the optimal position of ACT in the treatment of LACC. To critically appraise the OUTBACK trial and understand the consequences of its results, we used only randomized controlled studies (RCTs) on ACT in LACC that have been included in high-quality systematic reviews and meta-analyses. We calculated the pooled prediction intervals using a random effects meta-analysis of all published randomized studies including the OUTBACK trial. After combining the OUTBACK trial with the results of four previous randomized trials, the pooled hazard ratio for overall survival benefit of CCRT + ACT was 0.95 (95% CI 0.75; 1.20). The pooled hazard ratio of the four previous trials was 1.00 (95% CI 0.69; 1.44). The OUTBACK trial improved the precision of the pooled estimate, but the clinical heterogeneity and the consequent prediction intervals are still very wide, and with 95% reliability, we can expect that if the new study, using a similar approach to the ACT, on a randomly selected patient population from the presented five trials is conducted, its hazard ratio for overall survival after ACT would be between 0.47 and 1.93. In conclusion, there is an absolute need for further research in order to optimally define the position of ACT in the treatment of LACC.

## 1. Introduction

Each year, cervical cancer (CC) affects approximately 0.6 million women worldwide, with more than half of those unfortunately succumbing to the extent of the disease [1]. This high mortality to incidence ratio is at least partly a consequence of CC’s unequal global distribution. CC is the most commonly diagnosed gynecological cancer and the leading cause of cancer death among women in developing parts of the world [1]. The social weight of CC is increased by the fact that a majority of the women are being diagnosed at a relatively young age and with locally advanced disease [2]. Due to all of the above, CC constitutes a major global health and societal burden, with an underemphasized need to improve its well established and proven primary and secondary prevention, as well as timely and optimal treatment.

Standard treatment for locally advanced cervical cancer (LACC) remains the concomitant application of cisplatin chemotherapy and radiotherapy [3,4,5,6,7]. Nonetheless, after completion of primary CCRT, 30–40% of patients present with local or distant recurrence of the disease [3,4,5,6]. In an attempt to improve still unsatisfactory outcomes in LACC therapy, several treatment strategies were explored, including the application of concomitant polychemotherapy [8], higher doses of cisplatin [9], surgery following CCRT [10], neoadjuvant chemotherapy before CCRT [11], and adjuvant (consolidation) chemotherapy (ACT) after CCRT [12,13,14,15,16]. The latter has caused great turmoil because findings on the efficacy of ACT of LACC after the CCRT therapy were inconsistent, and the OUTBACK trial was expected to shed some light regarding the topic. Considering its results were negative, here we claim that OUTBACK trial should not be the last RCT undertaken regarding this topic but that further research is required [17]. Moreover, having in mind the above mentioned CC’s global distribution and societal impact, we presume facing two challenges when developing adjuvant chemotherapy in the field of LACC. In order to develop sustainable, widely applicable adjuvant chemotherapy, the same should consist of generic, easily obtained cytostatic drugs. Consequently, the first challenge is to be active and improve existing outcomes significantly, and the second one is to be affordable and with that, available to many underserved patients in the developing world.

The real question is what makes CC so special that adjuvant therapy does not work [18,19]? It certainly is a unique cancer, because it is preventable, detectable, and treatable. Perhaps its uniqueness lies in its resistance to adjuvant chemotherapy? Locally advanced stages of a vast majority of other cancer types, regardless of histological subtype, are effectively treated with adjuvant chemotherapy, which contributes to a clinically relevant longer overall survival (OS) [20,21,22,23,24,25]. On the other hand, when approaching recurrent or metastatic CC, it is implied to use chemotherapy as a treatment backbone [26,27]. The response rates achieved by standard chemotherapy regimens in a first-line adjuvant or metastatic treatment setting of breast, colon, and lung cancer do not exceed 50% [28,29,30,31]. The results of the first-line chemotherapy regimens used in the treatment of metastatic uterine cervical squamous cell carcinoma are similar [32,33]. Moreover, the true chemosensitivity of one tumor is defined in the neoadjuvant setting, where the reported response rates in CC range from 80–85% [11,34]. According to the above stated facts, it seemed justified to hypothesize that adjuvant or consolidation chemotherapy will have its benefit on CC as well.

Until 2020, there were only four published RCTs of CCRT + ACT efficacy in LACC compared to CCRT alone, with one inconclusive review and meta-analysis [13,14,15,16,35,36]. The two most recent meta-analyses were by Horeweg et al. One was published this year and it incorporated the results from the fifth RCT, the OUTBACK trial [17,18,19]. In general, current data and knowledge strongly discourages the use of adjuvant chemotherapy in LACC [19].

The objective of this review was to present the inconsistencies of previous studies, along with the OUTBACK trial in more detail, and to rethink whether its results provide an unambiguous and definite answer to the optimal treatment of LACC.

## 2. Materials and Methods

This review presents current state of knowledge regarding the use of adjuvant chemotherapy in locally advanced cervical cancer with critical appraisal of the OUTBACK trial. To critically appraise the OUTBACK trial and understand the consequences of its results, we used only the comparable studies that have been included in the high-quality systematic review by Tangjitgamol et al. [36] and meta-analysis by Horeweg et al. [18], who already assessed their risks of bias. We additionally searched for RCTs published after 5 September 2020, i.e., from the date covered by Horeweg et al.’s systematic review [18]. Eligible studies were randomized controlled trials of radiotherapy with concurrent chemotherapy followed by ACT in the treatment of LACC FIGO stage IB-IVA in women ≥18 years of age and ECOG performance status ≤2, with no neoadjuvant therapy and OS as the primary or secondary outcome. The outcome we focused on was OS because this was the OUTBACK trial’s primary outcome. In two studies, Lorvidhaya et al. [14] and Kim et al. [15], we had to calculate the hazard ratios (HR) by the Parmar [37] and Tierney [38] methods because they were not originally published, but in both cases, we checked the results of our calculations with the results obtained by Horeweg et al. [18]. We calculated the pooled estimates of HR using the random effects model with a restricted maximum likelihood method and weighted the studies inversely to their variances. We decided in advance that we would use the random effects model because the core of our hypothesis was clinical heterogeneity, although we erroneously expected that the five RCTs would be more methodologically homogenous. For the pooled estimate, in addition to confidence intervals (CI), we calculated the prediction interval (PI). We calculated all CIs and PIs at the 95% level. The number of RCTs was too small for the quantitative analysis, e.g., meta regression, of the possible causes of inconsistencies, and we performed only the qualitative synthesis. We performed the statistical data analysis and drew the forest plot using StataCorp 2019 (Stata Statistical Software: Release 16. College Station, TX, USA: StataCorp LLC).

## 3. Results

### 3.1. Previous Trials

**Figure 1:**
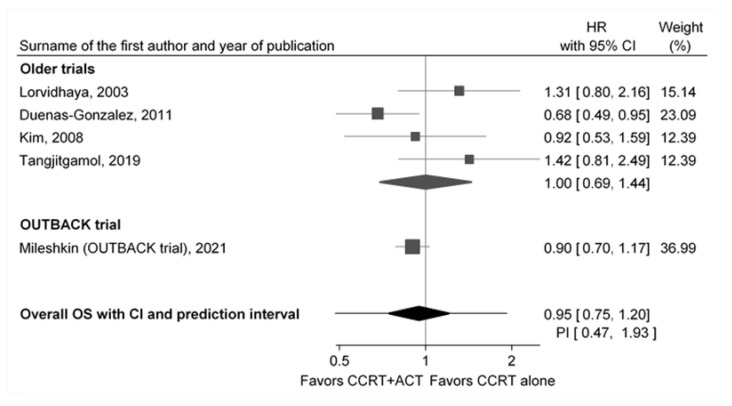
Forest plot of the effects of CCRT + ACT compared to CCRT alone on overall survival; gray squares represent each study hazard ratio (HR) for dying from any cause and whiskers represent 95% confidence intervals (CI); the size of the squares represent the weight of the study inverse to the study variance; black diamonds represent the pooled hazard ratio for older trials and for all trials (older and OUTBACK trial) calculated using a random effects model with a restricted maximum-likelihood method; whiskers from the lower “overall” diamond represent 95% prediction interval (PI); studies are sorted by the year of the end of enrollment.

The only RCT with statistically significantly better PFS (3-year PFS of 74.4% vs. 65%, HR 0.68) and OS (3-year OS with HR 0.68) in the CCRT + ACT arm (Table 1, Figure 1) was performed by Dueñas-González et al. [13]. This RCT, which controlled the balance of a relatively large number of predictive factors by minimization randomization with a concealed allocation of participants but no blinding, was performed in Mexico, Argentina, India, Panama, Bosnia and Herzegovina, Peru, Thailand, Pakistan, and Australia. Respecting the mortality-to-incidence ratio, from the perspective of the targeted population, this is the most relevant study on ACT in LACC conducted to date. The study had a reasonably short enrollment period of approximately two years, a relatively high proportion of patients in the CCRT + ACT arm who received at least one dose of ACT (86%), and the second-best ACT completion rate (77%). The key weakness of this otherwise well-designed study was the difference in the initial CCRT treatment between its two study arms: six cycles of cisplatin 40 mg/m^2^ in the CCRT alone arm, and six cycles of concurrent cisplatin 40 mg/m^2^ and gemcitabine 125 mg/m^2^ in the ACT arm of the study. In a later ACT protocol, patients received two additional cycles of cisplatin 50 mg/m^2^ with gemcitabine 1000 mg/m^2^. All patients received the same dose of RT, 50.4 Gy to the entire pelvic region in 28 fractions of 1.8 Gy/d, 5 days a week, over the 6 weeks of chemotherapy. Furthermore, after completion of CCRT, the majority of patients (93%) underwent low- or intermediate-dose rate brachytherapy (BCT) with cesium-137. A BCT dose of 30 to 35 Gy was delivered to point A to result in a cumulative dose of 80 to 85 Gy combining RT and BCT, and cumulative RT and BCT dose to point B (the pelvic wall) was 55 to 65 Gy. The ACT arm has started with adjuvant chemotherapy two weeks after BCT [13]. In addition to this regimen of RT and BCT, ACT arm also received the combination chemotherapy (cisplatin and gemcitabine) concomitantly with RT, unlike CCRT arm which received only monocisplatin concomitantly. Consequently, in this study, patients from ACT arm received different, combinational chemotherapy concomitantly with RT, as well as adjuvantly resulting in difficulties to define or measure impact of both of them on the final OS results. Higher toxicity rate of the combinational chemotherapy could have caused the difference in the discontinuation rate between these two study arms. In this sense, the dropout patterns in the Dueñas-González et al. study may have been somewhat different than in the other studies.

The oldest RCT was performed by Lorvidhaya et al. in Thailand, from 1988 to 1994, and it found, although not significantly, a better effect for CCRT alone (HR 1.42 was calculated by Parmar [37] and Tierney [38] methods) (Table 1, Figure 1) [14]. Randomization was stratified for the six study centers included, and consequently, two study arms were not perfectly balanced for the disease stage. Patients randomized to the CCRT + ACT arm had less frequent stage IIB and somewhat more frequent stage IIIB disease than the patients in the CCRT alone arm. Patients have received conventional RT which consisted of external RT and BCT. External RT was given to the whole pelvis in dose of 40–50 Gy with a midline shield to give the pelvic lymph nodes a dose of up to 50 Gy. A parametrium dose of up to 60–66 Gy was added to the involved side, depending on the extent of parametrial involvement, while BCT was given either high or medium dose rate, according to the standard in each center. The high-dose rate was 700–750 cGy at point A; two times per week for 2 weeks (four applications). The medium dose rate was a single application of 2500–2800 cGy to point A or two applications of 1400–1750 cGy to point A. The total dose at point A was 68–80 Gy. In other relevant characteristics, the patients in the two study arms were well balanced. Although the median age of patients enrolled in the Lorvidhaya et al. study was the second-highest compared to the other four RCTs, age above 65 was an exclusion criterion in this study. The distribution of age was not properly reported, nor is it an analysis of the possible moderating effect of age on OS. The most questionable part of this study was the chosen ACT protocol, monochemotherapy with 5FU, which many consider as not optimal for adjuvant therapy of LACC.

The smallest study with the longest enrollment period of seven years was conducted by Kim et al. in Korea from 1998 to 2005, with 78 patients in CCRT + ACT and 77 in the CCRT alone arm [15]. The study found no statistically significant longer OS and PFS in the CCRT + ACT arm (4-year OS of 70% vs. 67%, HR 0.92; 4-year PFS of 67% vs. 66%) (Table 1, Figure 1). Although the Kim et al. study had a randomization stratified for tumor stage, probably due to the relatively smaller samples, the final allocation resulted in a certain level of disbalance comparable to that from the Lorvidhaya et al. study (Table 1). In the initial CCRT arm, a relatively lower dose of cisplatin was administered: six cycles with 30 mg/m^2^. RT comprised of external irradiation to the whole pelvis of 41.4–50.4 Gy in 23–28 fractions plus high-dose rate (HDR) BCT (30–35 Gy in 6–7 fractions) to point A, together with a parametrial boost. One of the important limitations of the Kim et al. study was the low completion rate: 73% for CCRT alone, and 65% in the CCRT + ACT arm. However, the most important limitation is the fact that two out of the three cycles of chemotherapy based on the cisplatin and 5-fluorouracile (5FU) were given concomitantly with the external part of the radiotherapy. Therefore, this is not exactly the study of adjuvant chemotherapy, but more of a two types of concomitant chemoradiotherapy schedule.

The Tangjitgamol et al. study, conducted between 2015 and 2017 in Thailand, found no statistically significant benefit of ACT on OS (Table 1, Figure 1) [16]. Moreover, among the five studies compared, this study resulted in the least favorable results for ACT with 3-year OS of 69.5% in the CCRT + ACT arm vs. 80.1% in the CCRT arm and 3-year PFS of 63.4% in the CCRT + ACT arm vs. 66.6% in the CCRT arm (HR 1.26). The key limitations of this very well designed and executed RCT were its low completion rate (65%) and rather small number of randomized patients for a phase III trial, although rationally founded. While systemic recurrences were significantly lower in the ACT arm of the study, 5.4% vs. 10.1% (*p* = 0.029), defining the expected efficacy of ACT in the therapy of LACC, the OS HR was 1.42 (95% CI = 0.81–2.49; *p* = 0.221). This was the only RCT that masked the outcome assessment and the only one that used a standard six cycles of cisplatin 40 mg/m^2^ protocol as the CCRT in both study arms, while RT comprised of 45–50.4 Gy given in 25–28 fractions, 1.8–2 Gy/day, 5 days a week, and patients had high-dose rate BCT 6.0–7.5 Gy for 3–4 fractions.

### 3.2. Critical Appraisal of the OUTBACK Trial

#### 3.2.1. Study Overview

The OUTBACK was an international phase III trial of ACT after CCRT, compared to CCRT alone, as the primary treatment for LACC (ClinicalTrials.gov identifier: NCT01414608). Eligible patients were women with LACC ≥ 18 years of age, FIGO 2008 stage IB1 (only node positive), IB2, II, IIIB, IVA with ECOG performance status 0–2. The study enrolled 739 patients from USA and Canada, 165 patients from Australia and New Zealand, and 15 patients from China, Saudi Arabia, and Singapore. In the CCRT + ACT arm, 461 patients were enrolled, 83% completed the CCRT treatment as planned, 78% received at least one ACT dose, and 62% completed the ACT treatment as planned. In the control, the CCRT alone arm, 465 patients were enrolled, and 84% completed the CCRT treatment as planned. The CCRT was the same in both arms, consisting of five cycles of cisplatin and external-beam RT for five weeks, then intracavitary brachytherapy. ACT began four weeks after CCRT, with four cycles of paclitaxel and carboplatin. The primary outcome was OS at the fifth year from randomization, and the median follow-up was 60 months. The secondary outcomes were progression-free survival, adverse events and patterns of disease recurrence [17]. The analysis that has been presented so far was Kaplan–Meier curves and an unadjusted log rank test. The OUTBACK trial found no statistically significant differences in OS between patients allocated to the two study arms. OS after five years was almost the same in the CCRT alone arm (71%) and in the CCRT + ACT arm (72%). The difference was <1% (95% CI -6; 7%) [17]. HR for OS was 0.91 (95% CI 0.70; 1.18) and for PFS 0.87 (95% CI 0.70; 1.08), and adverse events of grades 3 to 5 occurred in 81% of patients in the CCRT + ACT arm compared to 62% in the CCRT alone arm during the first year after the randomization. Finally, the patterns of disease recurrence were similar. The OUTBACK trial was properly designed and well executed.

#### 3.2.2. External Validity

The OUTBACK trial’s targeted population was defined precisely but not too narrowly [17]. The study enrolled 33% of patients with FIGO 2008 stage IB1 (only node positive), IB2 or IIA tumors. Compared to the other RCTs, the OUTBACK population had a less advanced disease stage at the time of enrollment, which could have had an effect in favor of the null hypothesis of no ACT-relevant additional benefit to the effects of CCRT. The OUTBACK trial sample allocation was not proportionate to the population sizes in different countries. Furthermore, nonwhite patients in the OUTBACK trial’s ACT arm had two times higher odds for not even starting the targeted intervention.

#### 3.2.3. Internal Validity

OUTBACK was an open-label trial with no concealed allocation nor masking for the treatment assignment of participants or those delivering treatment, and with no blinded outcome assessment [17]. This could jeopardize the OUTBACK trial internal validity to a certain extent. The stratified randomization was used. Stratification was done for nodal status, participating site, FIGO stage, age and planned extended-field radiotherapy. However, randomization was performed before CCRT, so no stratification was conducted for completion of CCRT. The two study groups were well balanced at baseline in terms of age, ECOG performance status, geographical region, tobacco smoking, nodal involvement, extended field planned, FIGO stage, histology, and tumor diameter. Black participants were somewhat less prevalent in the CCRT + ACT arm. The planned initial treatment was the same in both arms, but the number of weekly cisplatin cycles was lower in the CCRT + ACT arm. This imbalance was accounted for by the sensitivity analysis, and no significant differences in OS or PFS in the CCRT + ACT arm were found between those who did and did not complete CCRT. In all other parameters of the initial CCRT treatment, participants from the two study arms were well balanced. No data was presented yet on the eventual differences between the two study arms in the treatment, other than the intervention of interest, especially of the treatment after disease progression, which could have influence on the OS results [40]. An important limitation of the study was the relatively low completion rate of the targeted intervention. ACT was administered in only 78% of participants assigned to the intervention arm, and 62% received all four ACT cycles as planned. In 2016, the OUTBACK trial protocol was amended to increase the sample size from 780 to 900, due to nonadherence with ACT, but at the 2021 Virtual ASCO Annual Meeting, from 4–8 June 2021, only the intention-to-treat analysis with Kaplan–Meier curves and unadjusted log rank test results were presented, with no sensitivity analysis or per-protocol analysis, which could help explain the effects of poor adherence with the ACT treatment. The duration of enrollment in the OUTBACK trial was six years.

### 3.3. Contribution of the OUTBACK Trial

After combining the OUTBACK trial results with the results of the four previous RCTs, using a random effects model with a restricted maximum-likelihood method, the overall HR for OS was 0.95 (95% CI 0.75; 1.20) (Figure 1). The pooled HR of the four previous studies was 1.00 (95% CI 0.69; 1.44). The OUTBACK trial, with its relatively large sample, markedly improved the precision of the pooled estimate. It also narrowed the PI from 0.23 to 4.28 in the previous four studies to the overall 0.47 to 1.93. However, the heterogeneity and the consequent PI are still very wide, and with the 95% reliability, we can expect that if the new study, with similar design, randomly selected from the population of the presented five RCTs, is conducted, the pooled HR for OS would be between 0.47 and 1.93.

## 4. Discussion

There are only five RCTs on ACT in LACC. All of them, including the OUTBACK trial, share some common weaknesses that could jeopardize their internal validity. The main limitation is the relatively low completion rate. No study was double-blinded, and only one properly masked the outcome assessment [16]. Only Dueñas-González et al. concealed the allocation and performed the adaptive randomization controlling for a large number of relevant prognostic factors [41]. Only the two newest studies had approximately comparable interventions [16,17], and the OUTBACK trial has not yet reported and controlled the post-ACT treatments that may have biased the findings on OS.

The OUTBACK trial was expected to establish a definite LACC treatment approach. Unfortunately, based on the results from previously published RCTs, including the two most recent by Tangjitgamol et al. and the OUTBACK trial which were two properly designed and conducted trials, the current state of knowledge of adjuvant chemotherapy in the treatment of LACC does not recommend its use in everyday clinical practice. Now, the question is: do we need more RCTs or not? Do we have definitive answers on this topic or not? Can we once and for all close the subject of adjuvant chemotherapy in LACC?

### 4.1. Predictive and Prognostic Factors That Could Have Caused the Inconsistencies

We have discussed different predictive and prognostic factors as the possible causes of the inconsistencies among these five RCTs in terms of ACT effects on OS. However, having in mind the wide predictive intervals we presented earlier, all these interpretations also should be read as the proposal of factors used as a helpful guide in identifying subpopulations that could derive benefit from ACT.

#### 4.1.1. Nonadherence

The ACT initiation and completion rates in the OUTBACK trail were low [42], but they were not relevantly lower than those in the Kim et al. [15] or Tangjitgamol et al. studies [16]. The OUTBACK and these previous two RCT completion rates were markedly lower than the 86% initiation and 77% completion of the two cycles of cisplatin and gemcitabine in Dueñas-González et al. [13] or the 92% completion of three cycles of oral 5-fluorouracil in the Lorvidhaya et al. study [14]. Undoubtedly, low adherence may jeopardize the internal validity of the trial if, as is certainly the case, it is not randomly distributed among participants, but with regard to this, the OUTBACK trial was not an outlier. What needs to be studied and understood are the differences in adherence between Kim et al. [15], Tangjitgamol et al. [16], and the OUTBACK trial [17,42] compared to Lorvidhaya et al. [14] and Dueñas-González et al. [13]. Tertiary, post hoc analysis of the OUTBACK trial found the highest multivariable, adjusted odds for not starting ACT in patients older than 60, non-Caucasian women, and patients with poor physical function self-rated on QLQ-C30. In the Tangjitgamol et al. study, 74% of all reasons for not even starting the ACT treatment were the patients’ or their physicians’ decisions [16]. Loss to follow-up, protocol violation, hematologic toxicity, and progression combined accounted for 26% of the reasons for not starting the ACT, while patients’ decline of further treatment was not occurring at all as the reason for incompletion of the initial CCRT treatment. Furthermore, one of the possible causes for the premature discontinuation of ACT could be the socioeconomic background and health insurance status of the patients. Hence, these are the parameters that also should be monitored in the OUTBACK trial and all future trials. The reported initiation and completion rates in OUTBACK, Kim et al., and Tangjitgamol et al. studies were markedly lower than in some observational studies [43,44,45]. In our personal practice, the adherence for completion of CCRT was 100%, while four to six cycles of ACT were received by 80% of patients [46,47,48].

#### 4.1.2. Stage of the Disease

Firstly, it is well known that the relative and absolute benefit of adjuvant chemotherapy is larger for more advanced stages of local disease irrespective of the type of tumor, cervix included [20,21,22]. Dueñas-González et al. found the better ACT effects in stage III or IV adenocarcinoma [41]. The comparable RCTs on patients with higher stages of LACC performed by Tangjitgamol et al. in 2019 found the significantly lower rate of systemic recurrences in the ACT arm (paclitaxel plus carboplatin) than in the CCRT arm (5.4% vs. 10.1%; *p* = 0.029), although no significant differences in overall or locoregional recurrences or three-year PFS or OS [16]. Comparably, the RCT performed by Dueñas-González found a significantly lower distant failure rate in the ACT arm (8.1% vs. 16.4%; *p* = 0.005) [13]. So did Tang et al., 2012 (14.3% vs. 23.6%; *p* < 0.005), but with one additional cycle of neoadjuvant cisplatin and paclitaxel in the ACT arm [49]. A recent meta-analysis by Horeweg et al. found the benefit of ACT after CCRT on distant-metastasis-free survival as well, although no significantly longer PFS or OS [18]. Moreover, the median tumor diameter in the Dueñas-González et al. trial was larger than in the OUTBACK trial and all three other RCTs. However, the Lorvidhaya et al. trial enrolled the largest proportion of patients with stage IIIB disease and found the second-worst effects of ACT on OS.

It is important to once again emphasize that the OUTBACK trial enrolled patients with Stage IB1 only in the case of a nodal positivity, which would, according to the current classification, upgraded them into Stage III1C, consequently with higher risk for both locoregional and distant failure [50]. However, from the available data, it is not clear how many of these patients could actually be upgraded nor type of the diagnosis of the nodal involvement. Hence, they are considered as patients with lower disease stage, alongside with patients with Stage IB2 and IIA. Although it represents a relevant difference compared to previous studies (33% of total patient population is more than in other RCTs), it will allow the authors of the OUTBACK trial to analyze the effects of ACT in the lower stages of LACC, which may prove particularly valuable from the perspective of our main conclusion. On the contrary, it should be considered a weakness of the OUTBACK trial. Namely, a higher proportion of patients with a lower stage of the disease could result in higher rates of noninitiation of ACT. It could motivate physicians and patients not to initiate ACT at all and/or not to complete the planned intervention, because the relative importance of toxicity is larger in less severe illness, and the perception of need for ACT may be lower in patients with a less severe disease stage.

Furthermore, the stage migration, in time and place, based on the diagnostic infrastructure, the quality of radiology and multidisciplinarity in general, is one of the most important factors in defining the outcomes of the patients enrolled in the RCTs, as well as in the everyday clinical practice. Generally speaking, older RCTs and trials that were conducted in less resourced medical environments tend to have under-staged patients and consequently worse outcomes. Following that statement, newer RCTs, especially the OUTBACK trial which is mostly conducted in well-resourced medical systems, should be more precise, and have less impact of potential stage migration on the real results of adjuvant chemotherapy in cervical cancer.

#### 4.1.3. Regional Differences

Given the sample allocation by country, the OUTBACK study can be considered the first international RCT conducted in developed countries. Due to the rather significantly large discrepancies between the relative sample and population sizes in particular countries and marked differences in mortality-to-incidence ratios among the three groups of countries, the OUTBACK trial results should probably be reported without the results from China, Saudi Arabia, and Singapore. Additional analysis of the Dueňas-González et al. trial has indicated differences in the ACT effects in different, less developed countries [41]. Due to discrepancies in incidence and mortality, disparities regarding the treatment availability across many developing countries, and considering the described allocation of the OUTBACK trial sample, new RCTs are absolutely needed in order to properly understand the possible effects of ACT globally.

#### 4.1.4. Treatment

Regarding the intervention, the most similar study or perhaps the only one similar enough to the OUTBACK trial was the Tangjitgamol et al. study [16]. It may seem confusing that these two studies found such different results, albeit in the end, they came to the same conclusion. In both studies, the cisplatin regimen in CCRT was changed to paclitaxel and carboplatin in ACT. The carboplatin-based regimen was shown to be noninferior to the cisplatin-based regimen in metastatic or recurrent CC [51,52], but the Kitagawa et al. study found significantly shorter OS in patients treated with paclitaxel and carboplatin if they had not received prior cisplatin. Initial CCRT in both the abovementioned trials were cisplatin-based, but still, in the available literature on ACT in LACC, there is no example of a paclitaxel-cisplatin ACT regimen to assess the possible effects of this switch to carboplatin. Therefore, special attention should be raised on the subject of the chemotherapy chosen for the OUTBACK trial. While four cycles of ACT are in line with what could be recommended in other oncology areas, especially after the CCRT part of the therapy, the intensity of ACT is not according to the widely accepted and used standards [53]. The usual dose of paclitaxel in a three-week schedule is 175 mg/m^2^, and the usual dose of carboplatin is AUC 6 [53]. Moreover, questionable is the decision to switch from the cisplatin used in the CCRT part of the protocol to the carboplatin in the ACT. Consequently, when you add two potentially detrimental things (low adherence in the ACT arm of the study and rather nonconventional dose intensity), you can argue why per protocol analysis is needed as well as why the OUTBACK trial should not be considered the definitive answer on the efficacy of ACT in the CC field.

#### 4.1.5. Duration of Enrollment and Follow-Up

The length of the study enrollment period is also a potential reason for looking deeper into the results of the study. Six years is a rather long period, when treatment patterns could change and influence the study results [54]. The OUTBACK trial’s enrollment period was no longer than in Kim et al. [15] or Lorvidhaya et al. [14], which lasted seven and six years, respectively, but it was three times longer than in Dueñas-González et al. [13] or in Tangjitgamol et al. [16], which lasted for two years. The OUTBACK trial had the second-longest follow-up period of 60 months. In addition to the fact that longer follow-up is a value in itself, it also is associated with many risks to the internal validity of OS as an outcome. Namely, the longer the follow-up is, the higher the frequency of additional therapy after the end of ACT, and this therapy was not yet described in the OUTBACK reports, nor were the overall survivals adjusted for its effects.

### 4.2. Future Directions

Taking into account that ACT is a cornerstone in the treatment of many solid cancers, that the results of chemotherapy in the treatment of metastatic CC are quite comparable to the results in other types of cancers, and that there is a rather weak level of evidence and a small number of RCTs performed in not so optimal conditions, we think that there is still no definitive answer regarding the efficacy and safety of CCRT + ACT on LACC. CCRT + ACT has a potential role to further improve control of the disease, especially a distant one. When designing new regimens for the successful treatment of LACC, we must take into consideration patient and disease specificities, treatment cost and feasibility, due to the fact that the majority of cases of CC are diagnosed in undeveloped countries. Furthermore, novel RCTs with properly designed, widely applicable, treatment strategy for assesment of adjuvant chemotherapy, should be carried out in regions from which majority of targeted population of patients come from, such as countries with lower income and socioeconomic status. We have to change the underserved title for CC, invest more in the ACT research, and publish more studies in well-defined populations. The results of the OUTBACK trial are not negative, i.e., HR 0.86 for PFS and HR 0.90 for OS with early and constant separation of the PFS and OS Kaplan–Meier curves, which defines the possibility that a significant number of patients do derive benefit from ACT. CC patients’ inherent specificities (low socioeconomic background, lower education status, less than optimal health insurance level, less adherence to the suggested therapy) do not help in our quest to solve the issue of the value of ACT. Nevertheless, all these problems should not discourage us but on the contrary, generate more and more well designed and founded trials globally that will, once and for all, define the position of ACT in the LACC treatment approach.

We cannot end this report without stressing once again the lack of investment in CC research, both clinical and basic. On ClinicalTrials.gov, as of 15 November 2021, there were 358 breast cancer registered studies not yet recruiting and not mentioning ACT, and 122 comparable CC studies, but 27 breast cancer studies that mention ACT and only two dealing with ACT in CC in the future. Moreover, when comparing to the breast cancer, the age-adjusted worldwide incidence of CC in 2020 in ≥20-year-old females was 22.1 per 100,000, which was 28% of the breast cancer incidence. However, due to the mortality rate of 12.1 in CC compared to 22.6 in breast cancer, the mortality-to-incidence ratio of CC is almost two times larger than in breast cancer (0.55 vs. 0.28, respectively). Additionally, the CC mortality rate is highly inversely associated with country income level. In low-income countries, the age-standardized mortality rates for CC and breast cancer are almost equal, and the incidence of breast cancer is only slightly higher, whereas in the high-income countries, the incidence of breast cancer is markedly larger than the incidence of CC in comparison to mortality (Table 2). In addition, it can be argued that not only are CC patients underserved, but also CC as a cancer in general. Regarding the total number of manuscripts published between 2016 and 2020 and indexed in MEDLINE (PubMed), reporting on the results of randomized controlled trials (RCT) and mentioning ACT in the title, abstract or keywords represented 2% of all RCT in CC, while representing 18% in breast cancer [55]. Unfortunately, this translates into slow development of the field, leading to unsatisfactory treatment outcomes, especially in LACC and, together with a lack of successful treatment options for metastatic disease, to an unacceptable mortality rate. Instead of traditional, variable-oriented analysis, we think that a future, person-oriented analysis such as finite mixture modeling or latent class analysis is necessary, which will subsequently lead to the recognition of subpopulations of patients with different profiles of predictive and prognostic factors and different expected ACT effects. In our opinion, such analysis is more than needed, especially in the era of precision oncology, as it represents a combination of the traditional approach based on population averages (including the prediction or prognosis modeling) and personalized oncology focused on the individual.

### 4.3. Limitations

The main limitation of our analysis could be considered that the results of the OUTBACK trial have not yet been published in their final form. In our manuscript, all interpretations of possible causes of inconsistencies of the OS in different populations were bivariable, while no predictive or prognostic factor truly exists in the isolation from other factors. The number of relevant RCTs was too small for valid multivariable meta-regression analysis, but the individual patient data should be analyzed in this way by the authors of particular trials. The OUTBACK trial authors conducted a multivariable analysis of the patients’ characteristics associated with not starting ACT [42]. The overall effects, i.e., differences in OS between subpopulations of patients, should be analyzed in a comparable way. We did not take into account RT parameters, although they may affect OS. To critically appraise the OUTBACK trial, we have compared it to the studies with quite different interventions. In our search of the number of manuscripts indexed in MEDLINE, and reporting the results of RCT mentioning and not mentioning ACT, we did not check whether each record really reported on ACT or just mentioned it in some other role. For this reason, all the figures we presented are exaggerated. However, there is no reason to believe this exaggeration is different between manuscripts on breast or cervical cancer, and our estimates may be considered the best-case scenario.

## 5. Conclusions

Due to the relatively small number of RCTs, their methodological diversities, particularly in terms of intervention, and after including the OUTBACK trial in the analysis, our conclusion is that its results should not represent the final verdict and close the subject of ACT in LACC. Moreover, there is an absolute need for further research in order to optimally define the position of ACT in the treatment of LACC.

## Figures and Tables

**Table 1:** Overview of included randomized controlled trials sorted by year of the end of enrollment.

	Lorvidhaya [14]	Dueňas- González [13]	Kim [15]	Tangjitgamol [16]	Mileshkin OUTBACK [17]
Year of publication	2003	2012	2008	2019	2021
Country	Thailand	Multiple ^a^	Korea	Thailand	Multiple ^b^
**Outcomes**					
The main result favors ACT	no	yes	no	no	no
Overall survival (HR)	1.41 ^c^	0.68	0.92 ^c^	1.42	0.90
(95% CI)	(0.79; 2.16)	(0.49; 0.95)	(0.53; 1.59)	(0.81; 2.49)	(0.70; 1.17)
**Randomization**	Stratified ^e^	Minimization ^d^	Stratified ^f^	Stratified ^g^	Stratified ^h^
**Concealed allocation**	no	yes	no	not clear/yes	no
**Masking/blinding**	no	no	no	outcome ass	no
**Patients**					
Number of patients randomized	230/233	259/256	78/77	130/129	461/465
Enrollment (start-end year)	1988–1994	2002–2004	1998–2005	2015–2017	2011–2017
Duration of enrollment (years)	6	2	7	2	6
Patient median age (years)	50/48 ^i^	45/46	58/57	49/50	46/45
Range of patient age (years)	<65	22–68/18–70	36–75/34–73	23–68/26–68 ^j^	21–99/22–88
**Disease**					
Stage (%)					
IB1 (all node+), IB2, IIA	0/0	0/0	0/0	0/0	33/33
IIB	43/50	62/61	67/75	65/62	43/43
IIIA	1/1	<1/<1	6/3	1/3	0/0
IIIB	55/49	36/37	22/17	31/35	24/24 ^k^
IVA	0/0	2/2	5/5	3/0	-
Median tumor diameter (cm)	n.a.	6/6	5/5 ^l^	5/5	5/5
Histology (%)					
Squamous cell carcinoma	90/88	93/94 ^m^	96/95	77/76	83/79
Adenocarcinoma	6/9	7/6	3/3	20/22	15/17
Adenosquamous carcinoma	1/0	-	1/3	2/2	3/4
Small-cell carcinoma	3/3	-	0/0	0/0	0/0
Positive pelvic lymph nodes	yes	n.a.	yes	yes	yes
Para-aortic lymph nodes >1 cm	yes	no ^n^	no	no	no
Previous chemotherapy or RT	no	no	no	yes ^o^	yes ^p^
**Intervention** (%)					
Completed CCRT	95	n.a.	73	80	83
Received at least one ACT dose	n.a.	86	n.a.	77	78
Completed CCRT + ACT	92	77	65	65	62
CCRT in control arm (cycle × DRUG mg/m^2^ or AUC)	2 × MIT 10 2 × FU 300 mg/day	6 × CIS 40	6 × CIS 30	6 × CIS 40	5 × CIS 40
CCRT in ACT arm	2 × MIT 10 2 × FU 300 mg/day	6 × CIS 40 6 × GEM 125	2 × CIS 20 2 × FU 1000	6 × CIS 40	5 × CIS 40
ACT protocol	3 × FU 200 mg/day	2 × CIS 50 2 × GEM 1000	1 × CIS 20 1 × FU 1000	3 × PAC 175 3 × CAR 5	4 × PAC 155 4 × CAR 5
**Follow-up**					
Median follow-up (months) ^q^	89	46	39	27	60

Data are presented in CCRT + ACT arm/in control CCRT only arm if not stated otherwise. Abbreviations: OS, overall survival; HR, hazard ratio; CI, confidence interval; CCRT, concurrent chemoradiation; ACT, adjuvant (consolidation) chemotherapy; RT, radiotherapy; n.a., not available; CIS, cisplatin; GEM, gemcitabine; FU, fluorouracil; PAC, paclitaxel; CAR, carboplatin; A, AUC; MIT, mitomycin C. ^a^ Mexico, Argentina, India, Panama, Bosnia and Herzegovina, Peru, Thailand, Pakistan, Australia; ^b^ Australia, New Zealand, USA, Saudi Arabia, Canada, China, Singapore; ^c^ HR was calculated by Parmar [37] and Tierney [38] methods; ^d^ Minimization using Pocock and Simon algorithm [39], balancing disease stage (IIB vs. III-IVA), tumor diameter (<5 cm vs. ≥5 cm), study center (not clear, probably 9 that is one per country), radiation equipment (cobalt-60 vs. linear accelerator), age (<55 vs. ≥55 years); ^e^ Stratified for six study centers; ^f^ Stratified for tumor stage; ^g^ Mixed block with stratification for disease stage (IIV vs. III-IVA) and histopathology (squamous vs. adenocarcinoma or adenosquamous carcinoma); ^h^ Stratified for nodal status, participating site, FIGO stage, age, planned extended-field radiotherapy; ^i^ Mean instead of median; ^j^ Interquartile range instead of range; ^k^ Including IIIB and IVA; ^l^ Estimated from categories (≤4; 4.1–6; 6.1–8; ≥8.1) weighted by frequencies; ^m^ Including squamous cell, poorly differentiated and adeno/squamous carcinoma; ^n^ Para-aortic lymph nodes >1 cm were exclusion criteria, but 2.3% in ACT arm and 4.7% in CCRT alone arm had at least one; ^o^ Previous chemotherapy was not an exclusion criterion, but all patients had newly diagnosed cervical cancer, so the previous chemotherapy/radiotherapy were allowed only for other cancers; ^p^ However, not for cervical cancer; ^q^ Rounded down to the last full month.

**Table 2:** Breast and cervical cancer epidemiology 2020, in ≥20-year-old females, age-standardized rates per 100,000 [55].

	Worldwide	Income Levels				Continent					
		Low	Low Middle	Upper Middle	High	Asia	Europe	Northern America	Latin America	Africa	Oceania
Incidence											
Breast	79.7	56.2	51.6	73.3	135.0	61.3	123.8	149.0	86.5	67.8	146.3
Cervix	22.1	39.7	28.2	21.2	13.9	21.1	17.7	10.2	24.7	42.7	16.8
Ratio cervix to breast	0.28	0.71	0.55	0.29	0.10	0.34	0.14	0.07	0.29	0.63	0.11
Mortality											
Breast	22.6	30.5	24.5	20.2	21.5	19.9	24.7	20.9	22.5	32.3	24.5
Cervix	12.1	29.0	17.7	10.8	4.2	11.7	6.3	3.5	12.6	29.4	7.7
Ratio cervix to breast	0.54	0.95	0.72	0.53	0.20	0.59	0.26	0.17	0.56	0.91	0.31
Mortality-to-incidence ratio											
Breast	0.28	0.54	0.47	0.28	0.16	0.32	0.20	0.14	0.26	0.48	0.17
Cervix	0.55	0.73	0.63	0.51	0.30	0.55	0.36	0.34	0.51	0.69	0.46
Ratio cervix to breast	1.93	1.35	1.32	1.85	1.90	1.71	1.78	2.45	1.96	1.45	2.74

## Data Availability

The authors confirm that the data supporting the findings of this study are available within the article.
